# Targeted Vaccination against Human α-Lactalbumin for Immunotherapy and Primary Immunoprevention of Triple Negative Breast Cancer

**DOI:** 10.3390/cancers8060056

**Published:** 2016-06-16

**Authors:** Vincent K. Tuohy, Ritika Jaini, Justin M. Johnson, Matthew G. Loya, Dennis Wilk, Erinn Downs-Kelly, Suparna Mazumder

**Affiliations:** 1 Department of Immunology, Lerner Research Institute, Cleveland Clinic, Cleveland, OH 44195, USA; jainir@ccf.org (R.J.); johnsoj1@ccf.org (J.M.J.); loyam@ccf.org (M.G.L.); mazumds@ccf.org (S.M.); 2 Department of Molecular Medicine, Cleveland Clinic Lerner College of Medicine of Case Western Reserve University, Cleveland, OH 44195, USA; 3 Shield Biotech, Inc., Cleveland, OH 44106, USA; dwilk@shieldbio.com; 4 Department of Anatomical and Clinical Pathology, Cleveland Clinic, Cleveland, OH 44195, USA; erinn.downs-kelly@path.utah.edu

**Keywords:** triple negative breast cancer, immunoprevention, immunotherapy, cancer vaccine, α-lactalbumin

## Abstract

We have proposed that safe and effective protection against the development of adult onset cancers may be achieved by vaccination against tissue-specific self-proteins that are “*retired*” from expression at immunogenic levels in normal tissues as we age, but are overexpressed in emerging tumors. α-Lactalbumin is an example of a “retired” self-protein because its expression in normal tissues is confined exclusively to the breast during late pregnancy and lactation, but is also expressed in the vast majority of human triple negative breast cancers (TNBC)—the most aggressive and lethal form of breast cancer and the predominant form that occurs in women at high genetic risk including those with mutated *BRCA1* genes. In anticipation of upcoming clinical trials, here we provide preclinical data indicating that α-lactalbumin has the potential as a vaccine target for inducing safe and effective primary immunoprevention as well as immunotherapy against TNBC.

## 1. Introduction

We have previously shown that α-lactalbumin vaccination mediates protection against the development of murine breast cancer in the absence of any detectable inflammatory changes in all normal non-lactating tissues examined [1]. Based on these results, we have proposed that vaccination of healthy, cancer-free, adult women against α-lactalbumin may provide safe and effective immunoprevention of breast cancer [2]. Since all current prophylactic vaccines target pathogens, several unique issues inherently arise regarding the overall feasibility of applying an autoimmune strategy for primary immunoprevention of breast cancer [3]. These issues include the following: (1) whether adult women would be immunologically responsive to human α-lactalbumin, (2) whether a history of lactation would create an insurmountable tolerance that would preclude generating effective immunity against α-lactalbumin, (3) whether α-lactalbumin is immunologically available in human breast tumors, and (4) whether expression of α-lactalbumin in normal non-breast tissues would predispose to systemic autoimmune complications.

Here we provide experimental data that directly address these concerns. We found that *in vitro* priming of peripheral blood mononuclear cells (PBMC) from healthy adult women results in frequencies of α-lactalbumin-specific proinflammatory T cells consistent with those associated with protection against murine breast tumors. We also found that the frequencies of T cells secreting interferon-gamma (IFNγ) and the level of protection from the development of breast tumors are virtually identical whether α-lactalbumin vaccination occurs in parous mice with a history of lactation and breastfeeding or in non-parous mice with no such history.

In addition, we experimentally confirmed the results of several database searches indicating highly significant overexpression of α-lactalbumin in human triple negative breast cancer (TNBC) [4,5]. This confirmation involved several experimental approaches including RT-PCR, Western blot, and immunohistochemical analysis of human TNBC tissues, as well as longitudinal visualization of α-lactalbumin gene expression during *in vivo* growth of human TNBC in immunodeficient mice. This *in vivo* visualization of α-lactalbumin gene expression was facilitated by measuring bioluminescence from growing human HCC1937 TNBC cells stably transfected with a lentivirus designed to express firefly luciferase under regulation of the human α-lactalbumin promoter. Finally, we found substantial published evidence indicating negative immunohistochemical staining for α-lactalbumin in 78 normal human tissues examined, thereby confirming the widely held view that α-lactalbumin expression in normal human tissues is confined exclusively to the lactating breast [6].

Thus far, our studies have resulted in the issuance of two US patents [7,8]. Collectively, our results support the view that α-lactalbumin vaccination has substantial potential for providing therapy against TNBC recurrence as well as primary immmunoprevention of TNBC, the most aggressive form of breast cancer and the most common variant occurring in women with *BRCA1* mutations, a high risk population with the greatest need for primary immunoprevention [9,10].

## 2. Results

### 2.1. In Vitro Priming of Human PBMC to Recombinant Human (rh) α-Lactalbumin

**Figure 1:**
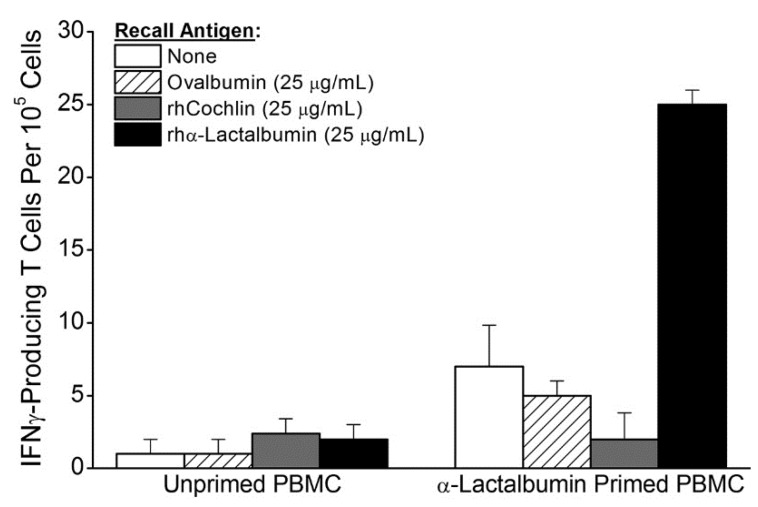
*In Vitro* Priming of Human peripheral blood mononuclear cells (PBMC) to rhα-Lactalbumin. Monocyte derived dendritic cells (DCs) prepared from PBMC taken from a 29 year-old healthy woman were loaded with purified rhα-lactalbumin and used to prime PBMC-derived T cells from the same donor. Recall responses to rhα-lactalbumin and the irrelevant control antigens ovalbumin and rhcochlin showed that 1 out of 20,000 cells were antigen-specific T cells. The observed α-lactalbumin responsiveness is representative of similar results obtained from several different women. Error bars indicate ±SD.

**Figure 2:**
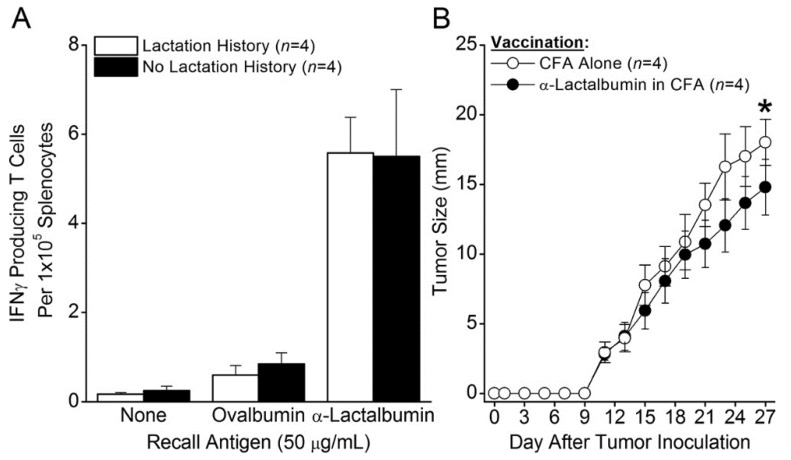
Effect of Lactation History on Induction of Tumor Immunity. Eight week old BALB/c female mice were mated and allowed to undergo one complete cycle of pregnancy, lactation, breastfeeding, and weaning. (**A**) Four weeks after weaning, parous mice and age-matched non-parous female mice with no prior lactation history were immunized with 100 µg α-lactalbumin in CFA. Four weeks after immunization, splenocyte frequencies of type-1 IFNγ-producing T cells were determined by ELISPOT analysis in recall responses to the priming α-lactalbumin immunogen. Frequencies of α-lactalbumin-responsive IFNγ-producing T cells that mediate immune protection against 4T1 tumors were virtually identical in mice with and without a prior history of lactation (mean ~1 per 20,000 splenocytes). (**B**) Four weeks after weaning, parous mice were immunized with α-lactalbumin in CFA or with CFA alone. Three weeks after vaccination, mice were inoculated with 1 × 10^4^ 4T1 breast cancer cells by subcutaneous injection in 100 µL PBS. Tumors were measured by Vernier caliper every other day until tumors reached 17 mm in any direction. Tumor progression analysis showed significant inhibition of 4T1 tumor growth (asterisk indicates *p* < 0.01) in mice immunized with α-lactalbumin compared to age-matched female mice immunized with CFA alone. Error bars show ±SE.

The feasibility of our vaccine strategy relies heavily on whether women have a T cell repertoire capable of mounting an effective proinflammatory immune response to human α-lactalbumin. To address this issue, we evaluated the size of the human female proinflammatory T cell repertoire induced by *in vitro* priming of PBMC to human α-lactalbumin. Monocyte derived dendritic cells (DCs) were pulsed with rhα-lactalbumin and used to prime enriched donor-derived T cells. The *in vitro* primed T cells were then tested for recall responsiveness in ELISPOT assays designed to determine the frequency of induced IFNγ-secreting α-lactalbumin-specific T cells. We found that healthy women have a T cell repertoire available for recruitment into a substantial proinflammatory T cell response to rhα-lactalbumin with an observed *in vitro* frequency of 1 per 20,000 cells (Figure 1). This frequency is similar to those obtained *in vivo* and shown to induce effective breast tumor immunity in BALB/c mice vaccinated against α-lactalbumin in complete Freund’s adjuvant (CFA; Figure 2a) [1]. Thus, we conclude that adult women likely have T cell repertoires capable of mounting a type-1 proinflammatory T cell response to human α-lactalbumin sufficient to induce effective tumor immunity.

### 2.2. Effect of Lactation History on Induction of Tumor Immunity

It is important to determine whether a history of lactation and breastfeeding creates an immunologically tolerant state that would prevent the induction of an effective tumor immunity targeted against α-lactalbumin. To determine the impact of lactation on the immune response to α-lactalbumin, we compared the T cell immunity and tumor protection induced in mice with and without a history of lactation and breastfeeding. Eight week old BALB/c female mice were mated and allowed to complete one full cycle of pregnancy, lactation, breastfeeding, and weaning. Four weeks after weaning, parous mice and age-matched non-parous female mice with no prior lactation history were immunized with rmα-lactalbumin in CFA. Four weeks after immunization, splenocyte frequencies of type-1 IFNγ-producing T cells were virtually identical in both groups of mice (~1 per 20,000 splenocytes; Figure 2a). Moreover, three weeks after vaccination with α-lactalbumin in CFA, parous mice with a history of lactation showed significant inhibition (*p* < 0.01) in the growth of inoculated 4T1 breast tumors compared to control mice vaccinated with CFA alone (Figure 2b). Thus, a history of lactation and breastfeeding had no discernable impact on either the frequencies of proinflammatory T cells or on the production of effective tumor immunity induced by α-lactalbumin vaccination.

### 2.3. α-Lactalbumin Gene Expression and Protein Detection in Human TNBC Cell Lines

**Figure 3:**
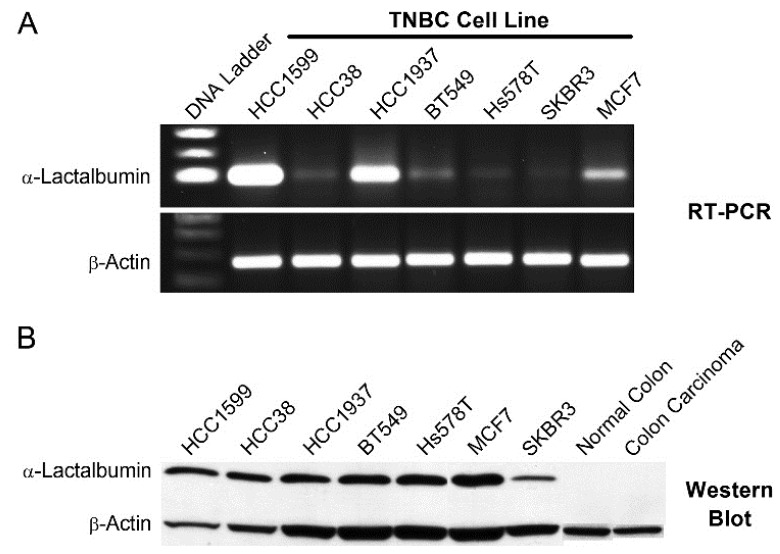
α-Lactalbumin Gene Expression and Protein Detection in Human Triple Negative Breast Cancer (TNBC) Cell Lines. (**A**) RNA was extracted from TNBC cell lines and underwent reverse transcription and 35 cycles of PCR gene-specific amplification for human α-lactalbumin and human β-actin. (**B**) For detection of α-lactalbumin protein in the same TNBC cell lines, lysates were electrophoresed on a 10% SDS polyacrylamide gel. Gels were blotted onto a polyvinylidene fluoride (PVDF) membrane and probed with antibodies specific for human α-lactalbumin and human β-actin. The data show variable levels of α-lactalbumin gene expression and protein production in most human TNBC cell lines.

The efficacy of α-lactalbumin vaccination is critically dependent on determining whether human breast tumors express α-lactalbumin and which breast tumors do so. Database searches repeatedly show highly significant overexpression of α-lactalbumin in human TNBC [4,5]. To further examine this issue, we evaluated α-lactalbumin gene expression and protein detection in human TNBC cell lines. RNA was extracted from several human TNBC cell lines, and after reverse transcription and 35 cycles of PCR gene-specific amplification for human α-lactalbumin and β-actin, the amplified products were visualized by agarose gel electrophoresis (Figure 3a). Western blot analysis was used to detect human α-lactalbumin protein in the same human TNBC cell lines. Lysates of cell lines were electrophoresed on a 15% SDS polyacrylamide gel, and the gels were blotted onto a polyvinylidene fluoride (PVDF) membrane and probed with antibodies specific for human α-lactalbumin and human β-actin (Figure 3b). RT-PCR amplification and Western blot analyses show varying degrees of α-lactalbumin gene expression and protein detection in human TNBC cell lines.

### 2.4. α-Lactalbumin Gene and Protein Expression in Human TNBC Tumors

**Figure 4:**
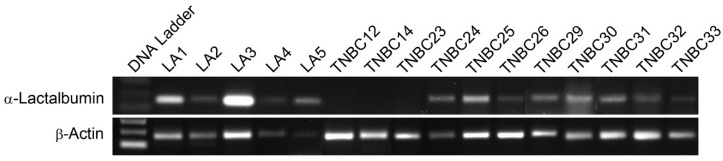
α-Lactalbumin Gene Expression in Human TNBC Tumors. RNA was extracted from 10 µm sections of formalin-fixed paraffin embedded TNBC tissue blocks for RT-PCR analysis of α-lactalbumin gene expression with visualization of the amplified products on an agarose gel. Lane 1 shows a DNA ladder. Lanes 2–6 show amplification from human lactating adenomas (LA) serving as positive controls. Lanes 7–17 show amplification from human TNBC primary tumors. Expression of α-lactalbumin (upper gel) was often comparable to expression of the β-actin housekeeping gene serving as an internal control (lower gel). α-Lactalbumin gene expression was detected in 8/11 (72%) primary human TNBC tumors examined.

**Figure 5:**
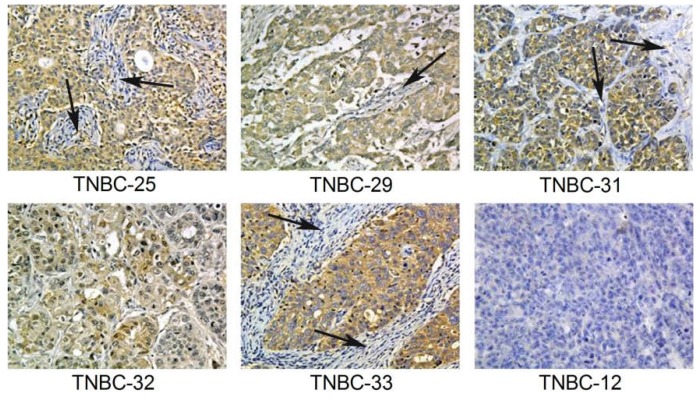
Immunohistochemical Detection of α-Lactalbumin Protein in Human TNBC Tumors. Immunohistochemistry for α-lactalbumin performed on formalin-fixed paraffin embedded human TNBC whole tissue sections showing that 5/6 (83%) tumors showed cytoplasmic immunoreactivity that was diffuse throughout the carcinoma with varying levels of intensity ranging from weak (TNBC-32) to moderate (TNBC-33). Note that the background stromal cells (arrows) are negative. TNBC-12 is negative for protein expression of α-lactalbumin, correlating with the absent gene expression (see Figure 4, lane 7). All sections are shown at 20×.

To assess α-lactalbumin gene expression in human TNBC tumor tissues, RNA was extracted from 10 µm sections of formalin-fixed paraffin embedded TNBC tissue blocks, and following reverse transcription, 35 cycles of gene-specific amplification were performed using human α-lactalbumin and β-actin specific primer pairs. Amplification products were visualized by agarose gel electrophoresis (Figure 4). α-Lactalbumin gene expression levels in 8/11 (72%) human TNBC were similar to levels measured in several lactating adenomas, a benign proliferation of breast tissue with lactational changes that occur in late pregnancy and the postpartum period. This 72% frequency of α-lactalbumin gene expression in human TNBC is similar to the frequencies reported in several searchable databases [4,5]. Using an α-lactalbumin-specific antibody, immunohistochemical analysis of formalin-fixed paraffin embedded human TNBC whole tissue sections showed that 5/6 (83%) human TNBC tumors examined had varying degrees of cytoplasmic immunoreactivity (Figure 5).

### 2.5. Visualization of α-Lactalbumin Gene Expression during In Vivo Growth of Human TNBC

**Figure 6:**
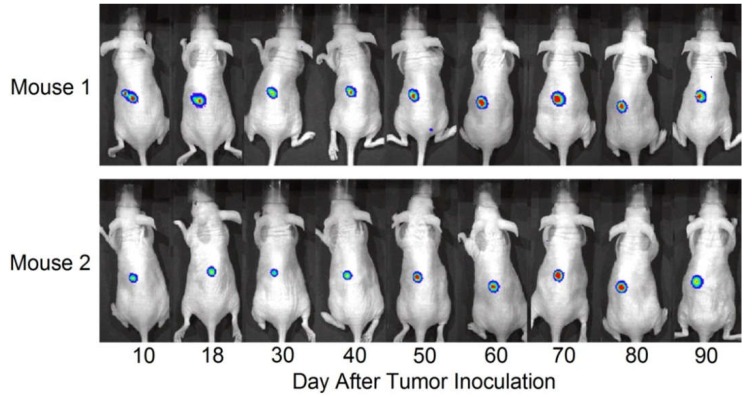
Visualization of α-Lactalbumin Gene Expression during *in vivo* Growth of Human TNBC. Human HCC1937-hlac-Luc cells express firefly luciferase under regulation of the human α-lactalbumin promoter. These stably transfected human TNBC cells were inoculated subcutaneously in the abdominal flanks of 6–8 week old immunodeficient, athymic, female NU/J mice and imaged regularly over a 90 day period for whole body full spectrum wavelength emission induced by injection with luciferin substrate. The stable bioluminescent signals obtained over 90 days in two tumor inoculated mice indicate that human TNBC tumors growing *in vivo* show stable constitutive α-lactalbumin gene expression over an extended period of time.

To visualize gene expression of α-lactalbumin in human TNBC breast tumors, HCC1937 TNBC cells were transfected with a lentiviral vector (hlac-Luc) designed to express firefly luciferase under regulation of the human α-lactalbumin promoter. The resultant HCC1937-hlac-Luc cells were inoculated subcutaneously in the abdominal flanks of 6–8 week old immunodeficient, athymic, female NU/J mice and imaged over a 90 day period of *in vivo* growth for whole body full spectrum wavelength emission induced by injection with luciferin substrate. Stable bioluminescent signals were obtained at all time points examined over 90 days in 2 tumor inoculated mice (Figure 6). We never observed any bioluminescence following injection of luciferin in NU/J mice bearing non-transfected wild type HCC1937 tumors or in NU/J HCC1937-hlac-Luc tumor bearing mice that were not injected with luciferin. Our data support the view that human TNBC tumors growing *in vivo* express α-lactalbumin in a stable manner over an extended period of time.

## 3. Discussion

In the current study we show that adult women have an available proinflammatory T cell repertoire capable of responding to rhα-lactalbumin at frequencies that are comparable to those observed following active immunization of mice with rmα-lactalbumin in CFA [1]. The observed frequency of 1 per 20,000 α-lactalbumin-specific type-1 proinflammatory T cells induced by *in vitro* priming is a frequency comparable to those obtained *in vivo* and shown to induce effective breast tumor immunity following active α-lactalbumin immunization of mice (Figure 2a) [1]. Thus, α-lactalbumin appears to be sufficiently immunogenic to induce effective tumor immunity in women. Moreover, if α-lactalbumin vaccination occurs in combination with an adjuvant capable of orchestrating a robust adaptive proinflammatory T cell response, any inherent immune tolerance to this self-protein will likely be overcome. Such adjuvants include CFA for use in animal experimentation and other adjuvants more suitable for human vaccination including the many DNA vaccines that induce IFNγ through CpG-Toll-like receptor 9 ligand interactions.

The likelihood of inducing proinflammatory immunity in women vaccinated with α-lactalbumin was further reassured when α-lactalbumin vaccinated parous mice with an established history of lactation and breastfeeding showed frequencies of proinflammatory T cells and tumor immunity indistinguishable from those occurring in non-parous mice with no such history. This outcome indicates that the sudden burst exposure of the adult immune system to massive levels of α-lactalbumin through the process of lactation does not irreversibly tolerize. These results indicate the feasibility of inducing a clinically relevant proinflammatory immune response to α-lactalbumin as long as the protein is properly and efficiently presented to the adult immune system.

We also found that α-lactalbumin gene expression occurs at substantial levels in the vast majority of human TNBC cell lines and in 72% of primary TNBC tissues examined. Moreover, the levels of α-lactalbumin gene expression occurring in TNBC cell lines and tumor tissues was similar to the levels detected in human lactating adenomas and correlated with Western blot detection of α-lactalbumin protein. In addition, we found that the α-lactalbumin gene is expressed constitutively and stably during both the early and later stages of TNBC tumor growth *in vivo*. Thus, the availability of α-lactalbumin protein in most human TNBC tumors makes it a suitable vaccine target for immunotherapy and primary immunoprevention of human TNBC.

It is well-established that α-lactalbumin gene and protein expression in normal tissues is confined to the breast during late pregnancy and lactation and is not detectable in any of 78 other normal human tissues examined [6]. Thus, it seems likely that this highly confined tissue expression precludes any systemic autoimmune complications in women vaccinated with α-lactalbumin. Thus, we believe that α-lactalbumin vaccination may be both safe and effective in preventing TNBC in women with *BRCA1* mutations who often have >80% lifetime risk for developing breast cancer with TNBC being the most common variant of this disease occurring in this high risk population [9,10].

Although it is not completely understood why α-lactalbumin is overexpressed in so many TNBC tumors, one explanation may be related to the loss of default inhibition that homeostatic levels of estrogen and progesterone signaling provide. Indeed, the rapid drop at parturition in production of estrogen, and perhaps more importantly progesterone, removes the impact of their homeostatic default inhibition of prolactin transcription that serves as the primary form of constitutive regulation of α-lactalbumin production [11,12,13,14,15]. Thus, we propose that breast tumors with deficient constitutive estrogen or progesterone signaling may be unable to provide sufficient default transcriptional inhibition of critical lactation proteins like α-lactalbumin. Experiments are ongoing to test this hypothesis.

Finally, it is worth reflecting on the aggressive nature of human TNBC tumors. This aggressiveness may best be appreciated by the ability of human TNBC tumors to grow in the complete absence of cognate signaling by the potent breast cancer growth factors, estrogen and progesterone. It seems ironic that the acquisition of such a prominent evolutionary advantage to grow without any need for readily available growth factors occurs with a tradeoff that the tumor may no longer be capable of sufficiently inhibiting α-lactalbumin synthesis, a tradeoff that may be exploited using α-lactalbumin targeted immune strategies.

## 4. Materials and Methods

### 4.1. Generation of Recombinant Human (rh) and Recombinant Mouse (rm) Proteins

The open reading frame cDNA nucleotide sequence for mouse α-lactalbumin (NCBI Reference Sequence: NM_010679.1) and human α-lactalbumin (NCBI Reference Sequence: NM_002289.2) were modified to ensure optimized protein folding and production in prokaryotic expression systems by substituting mammalian codons with more efficient prokaryotic sequences (Dapcel, Cleveland, OH, USA). The optimized DNA sequence was synthesized *de novo* and inserted into the NdeI-Bam HI sites of pET-3a expression vector (GeneArt AG, Regensburg, Germany, Darmstadt, Germany) thereby providing C-terminal 6xHis-tagged recombinant proteins. Plasmids containing these inserts were transformed in *E. coli* (Lucigen, Middleton, WI, USA). High level expression colonies were selected following induction with isopropyl β-D-1-thiogalactopyranoside (IPTG; Amresco, Solon, OH, USA) and were sequenced for confirming proper orientation and alignment. The 6xHis-tagged proteins were purified under denaturing conditions using nickel-nitrilo triacetic acid (Ni-NTA) affinity chromatography (Qiagen Sciences, Germantown, MD, USA). Prior to use, each protein was further purified by reverse phase high performance liquid chromatography (HPLC) to yield endotoxin-free protein [16]. Levels of endotoxin were <0.05 endotoxin units (<5 pg) per mg of recombinant protein.

### 4.2. In Vitro Priming of Human PBMC to rhα-Lactalbumin

Monocyte derived dendritic cells (DCs) were prepared from PBMC taken from a healthy, cancer-free, adult 29 year-old woman. Adherent cell selection was followed by culture in X-VIVO media (BioWhittaker, Walkersville, MD, USA) with 50 µg/mL rhGMCSF and rhIL-4 (Peprotech, Rocky Hill, NJ, USA). Six days after initiation of culture, DCs were pulsed with 75 µg/mL of purified rhα-lactalbumin, washed extensively 48 h later, and co-cultured with nylon wool purified naive T cells from the same donor at a ratio of 1:5 (DCs to T cells). After 72 h of co-culture, *in vitro* primed T cells and unprimed T cells from the same donor were enriched by passage through nylon wool and re-cultured in duplicate or triplicate with γ-irradiated (30 Gy) PBMC as feeder cells at a ratio of 1:10 (feeders to T cells) with 25 µg/mL rhα-lactalbumin, rhcochlin, or ovalbumin on ELISPOT plates (EMD Millipore, Billerica, MA, USA) pre-coated with mouse anti-human IFNγ capture antibody (Clone MD-1, eBioscience, San Diego, CA, USA). Frequencies of α-lactalbumin-responsive IFNγ-producing T cells were determined 48 h later using secondary biotinylated mouse anti-human IFNγ (Clone 4S.B3, eBioscience, San Diego, CA, USA). Final frequencies were determined by subtracting spots obtained in wells with 25 µg/mL ovalbumin from spots obtained in wells containing 25 µg/mL rhα-lactalbumin. Recall responses from unprimed T cells were consistently negligible.

### 4.3. Mice and Immunization

Eight week old BALB/cJ female mice were purchased commercially (Jackson Laboratory, Bar Harbor, ME, USA) and housed in microisolator cages in the Biologic Resources Unit of the Lerner Research Institute, Cleveland Clinic. Some mice were allowed to complete one full cycle of pregnancy, lactation, breastfeeding, and weaning prior to immunization with α-lactalbumin. Four weeks after weaning, parous female mice and age-matched non-parous female mice with no prior lactation history were immunized by subcutaneous injection in the abdominal flank with 100 μg rmα-lactalbumin in 200 μL of an emulsion of equal volumes of water and CFA containing 200 µg *Mycobacteria tuberculosis* H37Ra (Difco, Detroit, MI, USA). All protocols were pre-approved by the Institutional Animal Care and Use Committee of the Cleveland Clinic.

### 4.4. ELISPOT Analysis in Mice

Four weeks after immunization of mice with rmα-lactalbumin, splenocyte frequencies of type-1 IFNγ-producing T cells were determined by ELISPOT analysis. Duplicate or triplicate wells containing 2 × 105 splenocytes were cultured with 50 µg/mL rmα-lactalbumin or ovalbumin in ELISPOT plates (Millipore, Billerica, MA, USA) pre-coated with capture IFNγ antibody (clone AN-18; eBioscience, San Diego, CA, USA) in 200 μL/well total culture volume in DMEM (Mediatech, Manassas, VA, USA) supplemented with L-glutamine, penicillin, streptomycin, HEPES buffer (Invitrogen Life Technologies, Grand Island, NY, USA), and 10% fetal bovine serum (Hyclone, Logan, UT, USA). At 72 h of culture, wells were treated with biotinylated capture IFNγ antibody (clone R4-6A2; eBioscience, San Diego, CA, USA), and after overnight incubation and washing, spots were visualized by sequential treatment with alkaline phosphatase-conjugated streptavidin and 5-bromo-4-chloro-3-indolyl phosphate substrate (R&D Systems, Minneapolis, MN, USA). The reaction was halted after 10 minutes by repeated washing with double-distilled deionized H_2_O, and spots were developed and counted using an ImmunoSpot S6 analyzer with proprietary ImmunoSpot 5.1 software (Cellular Technologies Limited, Shaker Heights, OH, USA).

### 4.5. Evaluation of Mouse Breast Tumor Growth

Four weeks after weaning, parous mice were immunized with rmα-lactalbumin in CFA or with CFA alone as described above. Three weeks after vaccination, mice were inoculated with 1 × 10^4^ 4T1 breast cancer cells purchased commercially from the American Tissue Culture Collection (ATCC CRL-2539; Manassas, VA, USA) by subcutaneous injection in 100 µL PBS. Tumors were measured by Vernier caliper every other day until tumors reached 17 mm in any direction at which time all mice were humanely euthanized. Significance in tumor growth between groups was determined by analysis of variants (ANOVA).

### 4.6. α-Lactalbumin Gene Expression in Human TNBC

For analyzing gene expression in human tissues, RNA was extracted from 10 µm microdissected sections of formalin-fixed paraffin embedded human TNBC or lactating adenoma tissue blocks. For analyzing gene expression in human cell lines, RNA was extracted from human TNBC cell lines [17] obtained commercially including: HCC1599 (ATCC CRL-2331), HCC38 (ATCC CRL-2314), HCC1937 (ATCC CRL-2336), BT549 (ATCC HTB122), and Hs578T (ATCC HTB-126). Following reverse transcription, 35 cycles of gene-specific amplification were performed using purchased human α-lactalbumin and β-actin specific primer pairs (Applied Biosystems, Foster City, CA, USA) and the amplified products were electrophoresed on a 1.5% agarose gel.

### 4.7. α-Lactalbumin Protein Detection in Human TNBC

For Western blot detection of α-lactalbumin protein in human TNBC cell lines, cell lysates were electrophoresed on a 15% SDS polyacrylamide gel. Gels were blotted onto a polyvinylidene fluoride (PVDF) membrane and probed with a rabbit polyclonal antibody specific for human α-lactalbumin (#HPA029856; Sigma-Aldrich, St. Louis, MO, USA) and mouse monoclonal antibody for human β-actin (clone AC-15; Sigma-Aldrich, St. Louis, MO, USA) at dilutions of 1:100 and 1:8,000 respectively. Visualization of immunoreactivity was facilitated using horse radish peroxidase (HRP)-conjugated goat anti-rabbit or goat anti-mouse IgG (Southern Biotech, Birmingham, AL, USA). For immunohistochemical analysis, formalin-fixed paraffin embedded tissue sections at 5 μm underwent antigen retrieval in citrate buffer and were subsequently treated with a 1:100 dilution of the rabbit polyclonal antibody specific for human α-lactalbumin (#HPA029856; Sigma-Aldrich, St. Louis, MO, USA) followed by detection with HRP-conjugated goat anti-rabbit IgG (#4050-05; Southern Biotech, Birmingham, AL, USA).

### 4.8. Visualization of Human α-Lactalbumin Gene Expression during in vivo Growth of TNBC

A lentiviral vector (hlac-Luc) was generated to express firefly luciferase under regulation of the human α-lactalbumin promoter (Genecopoeia, Rockville, MD, USA). The human TNBC cell line, HCC1937, was transfected with the hlac-Luc vector, and stable transfectants were established by puromycin resistance. A total of 3–5 × 10^6^ stably transfected HCC1937-hlac-Luc cells were inoculated subcutaneously in the abdominal flanks of 6–8 week old immunodeficient, athymic nude NU/J female mice (Jackson Laboratory, Bar Harbor, ME, USA). Mice were pre-treated every other day for two weeks with intraperitoneal injections of asialo-GM1 antibody (eBioscience, San Diego, CA, USA) to deplete existing NK/NKT cells. Over a 90 day period, inoculated mice were imaged using the Xenogen in vivo imaging system (IVIS; Caliper Life Sciences, Hopkinton, MA, USA) 20 minutes after intraperitoneal injection with D-luciferin (Sigma Aldrich, St. Louis, MO, USA) at 150 mg/kg body weight.

## 5. Conclusions

Collectively, our findings and observations indicate the following:

- Healthy, cancer-free, adult women are capable of mounting a substantial proinflammatory T cell response to human α-lactalbumin.
- A history of lactation and breastfeeding has no impact on α-lactalbumin-induced immunity and on the protection that such immunity provides against the development of breast cancer.
- α-Lactalbumin is a breast-specific self-protein that is “*retired*” from expression in the aging female breast but is expressed in the majority of human TNBC.
- α-Lactalbumin vaccination may be most effective in providing therapy and primary immmunoprevention of TNBC, the most aggressive form of breast cancer and the form that predominates in women at high genetic risk due to mutations in their *BRCA1* genes [9,10].

## Abbreviations

The following abbreviations are used in this manuscript:

CFA	complete Freund’s adjuvant
DC	dendritic cell
ELISPOT	enzyme-linked immunospot
HPLC	high performance liquid chromatography
HRP	horse radish peroxidase
IFNγ	interferon-gamma
LA	lactating adenomas
hlac-Luc	lentiviral vector expressing luciferase regulated by the human α-lactalbumin promoter
Ni-NTA	nickel-nitrilo triacetic acid
PBMC	peripheral blood mononuclear cells
PVDF	polyvinylidene fluoride
rh	recombinant human
rm	recombinant mouse
TNBC	triple negative breast cancer
